# Mobile Crowd Sensing for Traffic Prediction in Internet of Vehicles

**DOI:** 10.3390/s16010088

**Published:** 2016-01-11

**Authors:** Jiafu Wan, Jianqi Liu, Zehui Shao, Athanasios V. Vasilakos, Muhammad Imran, Keliang Zhou

**Affiliations:** 1 School of Mechanical and Automotive Engineering, South China University of Technology, Guangzhou 510641, China; mejwan@scut.edu.cn; 2 School of Information Engineering, Guangdong Mechanical & Electrical College, Guangzhou 510515, China; liujianqi@ieee.org; 3 School of Information Science and Technology, Chengdu University, Chengdu 610106, China; zshao@cdu.edu.cn; 4 Department of Computer Science, Electrical and Space Engineering, Luleå University of Technology, Luleå 97187, Sweden; vasilako@ath.forthnet.gr; 5 College of Computer and Information Sciences, King Saud University, Riyadh 11451, Saudi Arabia; cimran@ksu.edu.sa; 6 School of Electrical Engineering and Automation, Jiangxi University of Science and Technology, Ganzhou 341000, China; nyzkl@sina.com

**Keywords:** mobile crowd sensing, traffic prediction, internet of vehicles, data aggregation, cloud computing

## Abstract

The advances in wireless communication techniques, mobile cloud computing, automotive and intelligent terminal technology are driving the evolution of vehicle *ad hoc* networks into the Internet of Vehicles (IoV) paradigm. This leads to a change in the vehicle routing problem from a calculation based on static data towards real-time traffic prediction. In this paper, we first address the taxonomy of cloud-assisted IoV from the viewpoint of the service relationship between cloud computing and IoV. Then, we review the traditional traffic prediction approached used by both Vehicle to Infrastructure (V2I) and Vehicle to Vehicle (V2V) communications. On this basis, we propose a mobile crowd sensing technology to support the creation of dynamic route choices for drivers wishing to avoid congestion. Experiments were carried out to verify the proposed approaches. Finally, we discuss the outlook of reliable traffic prediction.

## 1. Introduction

The Internet of Things technology is driving the evolution of Vehicle *ad hoc* Networks (VANETs) into the Internet of Vehicles (IoV) paradigm. IoV is an emerging field that crosses multiple disciplines, such as automotive, transportation, information and communications technology. The different perception of the vehicle in VANETs and the IoV makes these two scenarios differ fundamentally in the device, communications, networking, and services aspects. A vehicle in VANET is mainly considered as a node to disseminate messages among other vehicles, thus forming an inter-vehicle communication network. However, in the IoV paradigm, a VANET forms only a portion of the communication network. In addition to VANETs, dynamic vehicular mobile communication systems include inner-vehicle communication systems, such as Vehicle to Sensor (V2S), and communication with other entities, such Vehicle to Infrastructure (V2I), Vehicle to Human (V2H) and Vehicle to Internet [1,2,3].

Recently, cloud computing technology has emerged as a new information technology infrastructure for the fast developing IT industry. In [2], Liu proposed that, from the network perspective, the IoV system is a three-level “Client-Connection-Cloud” system, which includes the client, the connection and the cloud, respectively. Cloud-Assisted IoV (CAIV) is becoming a hot topic; it refers to representing physical system components, such as sensor-equipped vehicles and other devices in the cloud virtually, accessing (e.g., monitoring, actuating and navigating) those physical components through their virtual representations, and processing and managing the large amount of data collected from physical components in a scalable, real-time, on-demand, efficient and reliable manner [4,5]. Specifically, the integration of cloud computing techniques (e.g., virtualization, elastic re-configuration, and multi-tenancy of resources) with IoV techniques (e.g., vehicle social networks, and efficient big data analysis) appears to be a promising approach to advancing the state of the art, and allows previously unrealizable applications and services to be built, deployed, and managed effectively. Therefore, we believe that vehicular networking as a nascent form of IoV constitutes a very basic scenario of IoV. Recently, an increasing number of system technologies and system intelligence designs have been developed to make transportation cleaner, safer and more efficient. The IoV will play an important role in the clean traffic environment in the future. For example, reliable traffic prediction is very beneficial in saving travel time, reducing pollution, and improving traffic efficiency.

With the convergence of mobile communications and intelligent terminal technology, the transportation system is provided with a new approach in alleviating the traffic congestion through Mobile Crowd Sensing (MCS) technology, which is based on the power of various mobile devices such as smartphones and/or sensor-equipped vehicles [6,7]. In this sensing paradigm, participants, such as the drivers, can forward the traffic data obtained from mobile devices to the traffic monitoring system’s cloud. Then, traffic data analysis is carried out to inform drivers or related traffic authorities of the traffic situation.

Using vehicle communication, the roadside unit can ascertain a road’s status in real-time, and, along with the vehicular status, deliver these traffic data to the cloud, which in turn can estimate the average speed and other information. Unfortunately, these valuable traffic data is not being utilized by traffic departments, so no provision has been made for their effective transmission, storage and analysis. Currently, existing travel time prediction and vehicular dynamic route planning models do not analyze traffic data for drivers.

In this paper, we propose a method for utilizing data derived from connected vehicles to improve transportation efficiency. We present new algorithms for the timely estimation and prediction of travel times, and combine the results with accident prediction to support dynamic route choices for drivers to avoid congestion. In addition, this approach could potentially lead to building efficient large scale sensing applications by leveraging smartphones and/or sensor-equipped vehicles [8]. For example, instead of installing road-side cameras and loop detectors, one could collect traffic data and detect congestion levels using smartphones carried by drivers. Such solutions reduce the cost of deployment of specialized sensing infrastructure.

As previously described, the emerging technologies for traffic prediction are becoming a reality. In this paper, the goal of our research is to outline the current methods for traffic prediction and propose a novel method based on MCS technology. Our main features and contributions as follows:

- We discuss state-of-art traffic data collection and traffic prediction technologies in detail.
- We analyze the architecture of CAIV in a traffic analysis cloud environment and emphasize how to utilize the cloud to realize traffic prediction.
- We propose a novel algorithm based on MCS technology to predict traffic conditions.
- Also, we provide a qualitative comparison among three kinds of approaches, and we discuss our analysis and the field’s outlook.

The paper is organized as follows. The related works on CAIV, particularly from the perspective of traffic prediction, are presented in the next section. We then give the taxonomy of CAIV approaches and review traditional traffic prediction models used in both V2I and V2V communications. Following that, we propose a novel MCS approach to make traffic predictions. Subsequently, we carry out experiments to verify the proposed approach. Finally, we give some insights for reliable traffic prediction.

## 2. Related Works

In order to improve traffic conditions, some researchers have proposed traffic mitigation techniques. In this section, we review the state-of-art technologies on traffic data collection and traffic prediction.

### 2.1. Traffic Data Collection Technologies

In the past decade’s approaches, researchers usually made some measurements to collect traffic flow information. The most common intrusive and non-intrusive detection technologies are loop detectors and road-side cameras, respectively. A loop detector is buried underground and detects the pressure exerted by vehicles to count the number of vehicles passing over it. Traffic cameras not only count the number of vehicles, but can also identify plate numbers. These two approaches incur enormous infrastructure deployment costs, but they have been widely used in transportation systems for traffic flow monitoring. Once a slow traffic flow or an unexpected standstill is detected, the traffic situation regarding the particular road may be published dynamically on nearby billboards to inform drivers.

In recent years, On-Board Equipment (OBE) is being used to detect the status of vehicle using vehicular sensors. A GPS receiver can obtain the location; the speedometer can measure the vehicle’s speed; the odometer can obtain the distance travelled within an interval and various other inner sensors can obtain information about the vehicle’s condition. These traffic data can be delivered to a data center though a cellular network.

However, VANETs introduced a novel, more timely and interactive way of collecting traffic data. A VANET uses vehicles and/or smartphones as mobile nodes in a mobile *ad hoc* network to create a mobile network [9]. In this manner, Roadside Equipment (RSE) deployed at strategic locations can exchange information with smartphones carried by the drivers. RSE and proximate smartphones are interconnected and share traffic information (e.g., traffic congestion levels). Vehicles outside the range of any RSE may still be connected to the rest of the vehicle and infrastructure network via neighboring vehicles. This network can generate accurate real-time traffic information in great detail, based on which some fundamental traffic problems regarding efficiency can be addressed from a brand new perspective.

### 2.2. Traffic Prediction Technologies

#### 2.2.1. Travel Time Aggregation for Traffic Prediction

For the travel time aggregation process, communication channels are established between dynamic mobile systems based on vehicles and RSE units. In [10], Lochert *et al.* tackled the aggregation problem for the specific case of travel time data supporting road navigation decisions. Essentially, the travel time aggregation, including landmark- and hierarchical landmark-based aggregation, is achieved through compressing all available information on all possible paths between two landmarks to a “virtual” link connecting them. The basic idea of the aggregation scheme is based on landmarks such as junctions and intersections. Landmarks are defined on multiple levels of hierarchy in the road network. At the highest level, these are junctions of the main roads or highways, while lower levels include higher level landmarks and an increasing number of smaller street intersections. The lowest level is a representation of the full road network.

With the support of V2I communication, vehicles passing a road segment make an observation of the current travel time between two neighboring landmarks. This information is subsequently distributed to nodes within their close surroundings. It is then used by vehicles to calculate travel times between landmarks of the next higher level, thereby summarizing the travel times in the area. The details of landmark-based aggregation were analyzed in [11].

In order to perform hierarchical aggregation, landmarks are assigned a level in a hierarchy. Landmarks of a higher level are also members of all the lower levels. The destination of a trip will not always be a high-level landmark position. Nevertheless, the aggregated information can of course be used for route planning. In order to plan a route, a navigation system “fills in” the missing information between the final destination and close-by landmarks by using standard travel times hardcoded in the map data. This is reasonable, because a final decision on the last part of the route is not yet required at this stage—it is sufficient if a good choice for the immediately upcoming routing decisions can be made. As the vehicle approaches its destination, the route can be updated and refined as more detailed information becomes available.

#### 2.2.2. Spatio-Temporal Correlations for Traffic Prediction

A vehicle in a VANET not only connects the RSE, but also connects to other vehicles. Connected vehicles and roadside infrastructure can generate some traffic data, such as vehicular spatio-temporal trajectories, which allow a brand new perspective on addressing some key issues of traffic prediction, including: (a) how to accurately aggregate traffic data and predict future traffic conditions using the VANET; and (b) how to improve efficiency of traffic management by mining huge amounts of traffic data. In [12], Min *et al.* presented a new method for real-time road traffic prediction with spatio-temporal correlations. The method takes into account the spatial characteristics of a road network in a way that reflects not only the distance but also the average speed of the links. In [13], Li *et al.* proposed a real-time and reliable communication architecture for connected vehicles based on traffic responsiveness, a field theory model based on connected vehicles, and networked vehicle routing algorithms. In this study, a new method for traffic prediction was proposed through the combination of temporal and spatial traffic flow data (e.g., volume, density, and speed), which was simultaneously based on tensor feature regression. In [13], Wan *et al.* gave some insights from the point of view of context-aware, and carried out a simplified experiment for traffic prediction.

Even if the areas of interest are not covered by RSE, we can still carry out traffic predictions. The vehicle can cache traffic data in its memory and, once it passes by a RSE, it will transmit cached traffic information such as its origin, destination, and vehicle trajectory data, to the RSE. A lot of useful information, such as volume, speed, density, acceleration/deceleration rates, and travel times of upstream segments, can be processed by appropriate data analysis algorithms. This information can then be correlated with traffic data obtained from upstream/downstream RSE and traditional loop detectors. A mathematical relationship can then be established using the tensor regression method of [14]. The RSE mines the trajectory and loop detector data and continuously provides estimates and predictions on the state of traffic in areas not covered by RSE.

#### 2.2.3. MCS Paradigms in Transportation

Transportation is an obvious application area for MCS. Recently, some crowd sensing experiments were carried out to determine traffic congestion levels, traffic delays, and road condition problems (e.g., potholes). There are several notable examples:

- *MIT’s CarTel project* [15]. Both mobile devices and custom-built on-board telematics devices were used to gather information about vehicles’ location and speed. The CarTel system can estimate road travel times using a combination of historic and real-time information. These estimates are used in detection of congested road segments and in real-time route planning to minimize expected travel times. In this case, the energy consumption of the mobile devices is reduced by using inaccurate but less power-demanding positioning.
- *Microsoft Corp.’s Nericell project* [16]. This project focused on traffic in developing regions, with experiments conducted in Bangalore. In particular, emphasis was placed on the detection of potholes in roads, based on particular patterns of acceleration observations, and traffic congestion, where microphones were used to detect honking. The project introduced the notion of triggered sensing, in which an observation from a less energy-consuming sensor (e.g., an interesting GSM location) is used to activate a more power-hungry but accurate sensor (e.g., the GPS).
- *The University of California, Berkeley and Nokia Corp.’s Mobile Century and Mobile Millennium projects* [17]. The velocity of traffic in San Francisco was monitored using GPS-enabled mobile phones. Privacy was enforced through the use of spatial sampling that produced anonymized observations when crossing so-called virtual trip lines.
- *ParkNet* [18]: An application that detects available parking spots in cities using smart phones combined with ultrasonic sensing devices installed on vehicles.
- *Queen’s University (Canada)’s CrowdITS project* [19]. An ongoing effort to develop a crowd-based sensing system that uses both GPS logging and voice commands to gather information about congestion, and use the information for the real-time computation of congestion-free routes.

These projects have shown the feasibility of using GPS equipped mobile devices in sensing traffic situation. They indicate that even with a relatively low penetration of mobile devices, it is possible to get more detailed information and broader coverage of the traffic situation than solely with stationary sensors. Moreover, the experiments given rise to a variety of approaches for noisy GPS data processing and verification. Still, the central focus of these experiments has been on private vehicles and the route choices of individual users. However, the superiority of cloud-assisted MCS for transportation has not been studied. In this paper, we first review geographical data aggregation, and then propose a specific algorithm to realize reliable traffic prediction.

## 3. Cloud-Assisted Internet of Vehicles

As mentioned above, RSE deployed at strategic locations can exchange traffic data with vehicles [20,21,22]. V2I connectivity is critical to avoid or mitigate the effects of road accidents, and to enable the efficient management of intelligent transportation systems [23].

Recently, a few research projects conducted studies on the combination of cloud computing with vehicular networks. In [24], researchers proposed architectures of vehicular clouds, vehicles using clouds, and hybrid clouds. In [25,26], a hierarchical cloud architecture for vehicular networks was introduced, and the proposed architecture included a vehicular cloud, a roadside cloud, and a central cloud. Mobile Cloud Computing (MCC) technology, with its features of scalability and virtualization, can handle massive computing, storage and software services in a flexible manner [27,28]. The integration of IoV and MCC can promote the development of cost-effective, scalable transportation systems. CAIV is a promising approach that highlights some emerging applications and services, and it is hoped that a number of strategies for improving traffic efficiency and road safety and enabling a clean traffic environment will be introduced through this approach.

From the standpoint of the service relationship between cloud computing and vehicular networks, the architecture of CAIV can be divided into three primary architecture types: Vehicles to Clouds (VTC), Vehicles as Clouds (VAC), and Vehicles with Clouds (VWC). With MCC support, intelligent transportation systems can provide more elastic services, and even facilitate traffic prediction. In this paper, we analyze the service relationship between cloud computing and IoV, and mainly focus on how to utilize the traffic cloud to achieve traffic prediction. For VTC, vehicles can access cloud services from gateways deployed along the roadside infrastructure. VAC is composed of a set of connected passengers and/or vehicles, initially located in the same area as other users. Subsequently, they may opt to allocate their computing resources to other users, forming datacenters.

**Figure 1:**
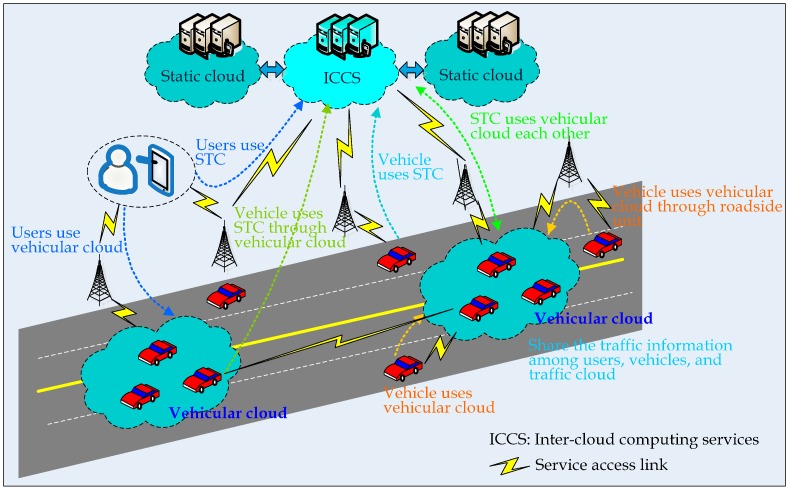
Information interaction for VWC.

Figure 1 shows the information interaction for VWC [29]. The ultimate goal of CAIV is to combine the features of VTC and VAC to serve the role of vehicle as infrastructure and end users simultaneously. In order to provide collaborative computing, the VWC architecture includes two clouds that work in tandem, a static and a dynamic cloud. The static cloud is a collection of stationary machines placed in a datacenter, while the dynamic cloud uses vehicles as cloud resources that add to the total computing capacity. Through the integration of the stationary cloud with the vehicular cloud, VWC has a great potential to afford more flexibility and services. The vehicular cloud, having broad sensing capabilities through vehicular sensor networks, can provide particular resources over the Internet to either the stationary cloud or the end users. By doing so, real-time road conditions can be assessed and published with the timely seamless information due to the mobility of vehicular cloud. The vehicle itself can have a connection with neighboring vehicular clouds to use services and applications over the Internet.

## 4. MCS for Traffic Prediction by VWC

MCS, as an emerging category of internet-of-things applications, leverages the sensors and computing power in mobile devices opportunistically to sense environmental conditions. In this paradigm, we achieve abundant cloud services by using V2V, V2I, V2H and V2S interactions to form a VWC architecture. The following describes the sensing methods and service process.

**Figure 2:**
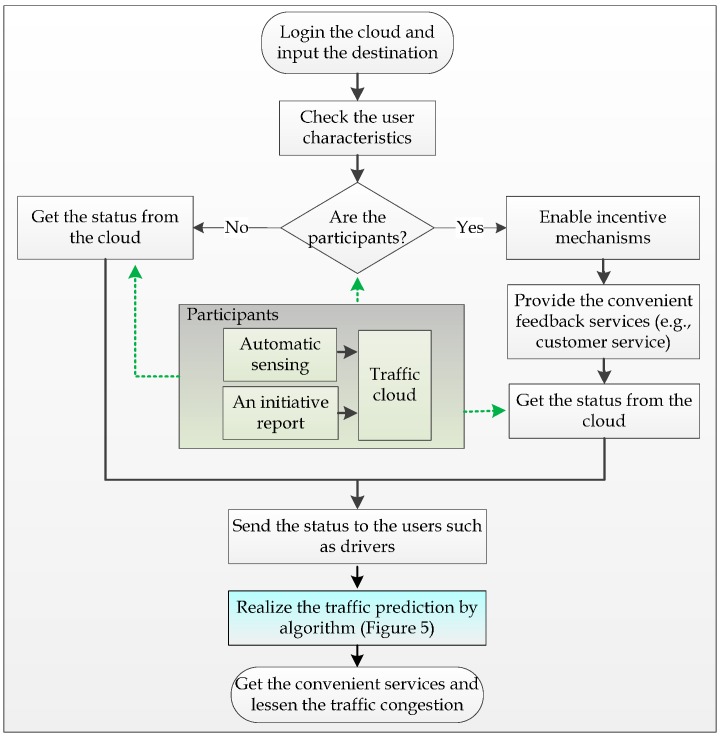
Flowchart of a cloud-assisted MCS traffic congestion control algorithm.

- *Automatic Sensing and Uploading Approaches*: According to the Mobile Century and Mobile Millennium projects, the results suggest that a 2%–3% penetration of smartphones in the driver population is enough to provide accurate measurements of traffic conditions. Therefore, at the early stage of the project implementation, we can make use of administrative means to obtain the participation of, for example, taxi drivers for the purposes of the experiments. Smartphones carried by taxi drivers can periodically forward data (e.g., *mobileId, location, speed, and direction*) to the traffic cloud through the mobile network. The duration of the period should be a tradeoff between energy consumption, data traffic and data reliability.
- *Service Process*: Figure 2 shows the logic flowchart of a cloud-assisted MCS traffic congestion control algorithm.

The drivers or passengers can quickly obtain traffic congestion levels by various smart terminals, such as smartphones, PDAs, or sensor-equipped vehicles. If a service request is derived from the participants, the system will automatically enable the incentive mechanism. The algorithm pseudocode for the service process is given in Algorithm 1.
**Algorithm 1**.**input:**  *T*: The sample period;  *s_d_* : The destination station**begin** Set the weight parameters α,βand γ; Construct all values of *d_i,j_* and *q_i,j_* according to the distances among stations; *T_c_*←current time; *T_l_*←*T_c_*; **while** conditions are satisfied **do** **if** (*T_c_*- *T_l_*≥*T*) **then**  Construct all values of *v_i,j_* and *r_i,j_* which are obtained from cloud servers;  Construct the weight *W* using Equations (3) and (4);  Get the next station *s_next_*;  Obtain and report the shortest path from *s_next_* to *s_d_* using Dijkstra’s algorithm;  *T_c_*←current time; *T_l_*←*T_c_* **else**  *T_c_*←current time; **endif**  **endwhile****end.**


**Figure 3:**
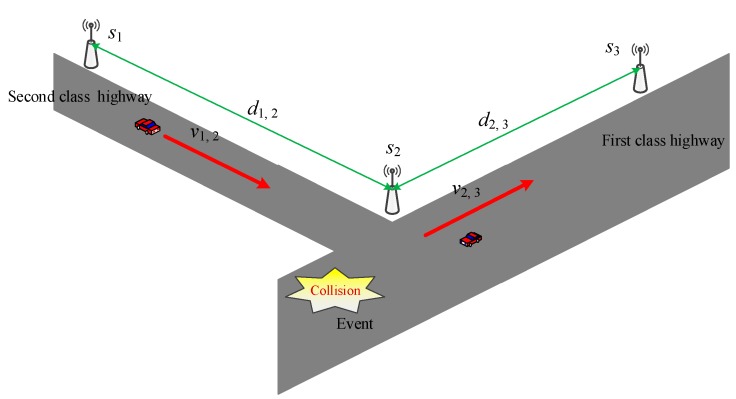
Traffic prediction based on VWC.

We further study the algorithm to realize traffic prediction with MCS technology. Let us assume the network under consideration has *N* stations, *s*_1_, *s*_2_, …, *s_N_*. The stations are usually deployed at intersections or junctions; the detailed diagram is shown in Figure 3. *R* (*s_i_*, *s_j_*) represents road segment from station *s_i_* to station *s_j_*. The distance between *s_i_* and *s_j_* is denoted by *d_i,j_*, which remains constant after the corresponding stations have been deployed. The variable *q_i,j_* is used to express the quality of the road, the value which lies in [0,1]. We set the value as follows:

(1) $$q_{i,j}=\{\begin{array}{c} 0,\;R\left(s_{i},s_{j}\right)\;is\;a\;first\;class\;highway;\;\\ 0.5\;R\left(s_{i},s_{j}\right)\;is\;a\;second\;class\;highway;\\ 1,\;R\left(s_{i},s_{j}\right)\;is\;a\;third\;class\;highway;\;\\ +\infty ,\;R\left(s_{i},s_{j}\right)\;can\;not\;be\;used.\; \end{array}$$

Therefore, as shown in the figure, the quality values of roads *R*(*s_1_*, *s_2_*), *R*(*s_2_*, *s_3_*) are 0.5 and 0, respectively. The variable *r_i,j_* is used to express the existence of an event that causes congestion. We call such an event an adverse event; a collision is typical such event. The value of *r_i,j_* is assigned as follows:

(2) $$r_{i,j}=\{\begin{array}{c} +\infty ,\;an\;adverse\;event\;has\;taken\;place;\\ 0,\;otherwise.\; \end{array}$$

The average road speed *v_i,j_* is derived in the traditional manner, *i.e.*, by dividing the distance *d_i,j_* from station *s_1_* to station *s_2_* by the vehicle travel time. This method is simple, but does not consider vehicle parking. If the driver goes shopping and the vehicle parks in a parking lot between station *s_1_* and station *s_2_*, the shopping time is accumulated with the travel time, so the value of *v_i,j_* is inaccurate.

In Algorithm 1, we can obtain the real-time speed using speedometer measurements in a specific period *T*. If the vehicle is located on the road, which can be verified using a GPS receiver and map matching software, we consider the speed value to be qualified at that moment. The average speed *v_i,j_* is the expectation of all qualified real-time speed values. It is underlined that, when the vehicle does not lie on a given road, the corresponding real-time speed value must be discarded. In the above-mentioned situation, speed measurements during shopping time should be discarded.

Subsequently, we construct a weighted directed graph *G* = (*V*, *E*, φ) as follows. Let *V* = (*s*_1_, *s*_2_, …, *s_N_*), and *E* = {(*s_i_*, *s_j_*) |, there exists a direct path between *s*_1_ and *s_j_*}, and φ be a function: *E*$\to R^{+}$ such that:

(3) $$\varphi \left(s_{i},s_{j}\right)=\frac{\alpha }{\sqrt{v_{i,j}}}+\beta d_{i,j}+\gamma q_{i,j}+r_{i,j}$$

where α,β,γ are the prescribed weights, and $\varphi \left(s_{i},s_{j}\right)$ is assigned to be +∞ if the value of *v_i,j_* is detected to be zero. We then obtain a weighting matrix $W_{N\;\times \;N}$ such that:

(4) $$W_{i,j}=\varphi (s_{i},s_{j})$$

If we want to obtain a path plan to the destination from any given location, we need to reach the next station *s_next_* first. Then, we obtain a routing from the station *s_next_* to the destination *s_d_*. Also, we may form an updated version of the path within a given period *T*. In other words, we obtain a new path every *T* seconds.

In order to reach this goal, we first set the parameters α,β,γ, the period *T*, the destination station *s_d_*, the distances and the road quality between stations. Then together with the information about vehicle speeds and events between stations, which is obtained from the cloud server, we obtain a weighting matrix *W* for the paths between stations and thus we form a directed weighted traffic network. Now, when asked to give the optimum path to a destination *s_d_*, we first reach the next station *s_next_* and compute the desired path from *s_next_* to *s_d_*.

When the traffic network status changes at higher frequencies, a shorter period period *T* must be set. When we set *T*, we also need to consider the ability of the cloud servers. Normally, we can set *T* = 300 seconds. The values of the parameters α,β,γ  are then prescribed according to practical needs. If we ignore the speeds of vehicles or the quality of the road, then α and γ can be set to be zero, respectively. Nornally, we must consider the distances, so the parameter β should be positive.

The pseudocode of the algorithm is described in Algorithm 1. Let *V* and *E* be the set of the vertices and the edges of a given network, respectively. The original Dijkstra algorithm does not use a min-priority queue and runs in *O*(|*V*|^2^) [30]. However, the same algorithm based on a min-priority queue implemented by a Fibonacci heap runs in *O*(|*E*| + |*V*|log(|*V*|)) [31]. Therefore, it can be seen that in each loop, the optimum routing path is available in *O*(|*E*| + |*V*|log(|*V*|)) once the traffic network is constructed.

## 5. Simulation and Experiment

### 5.1. Simulation

**Figure 4:**
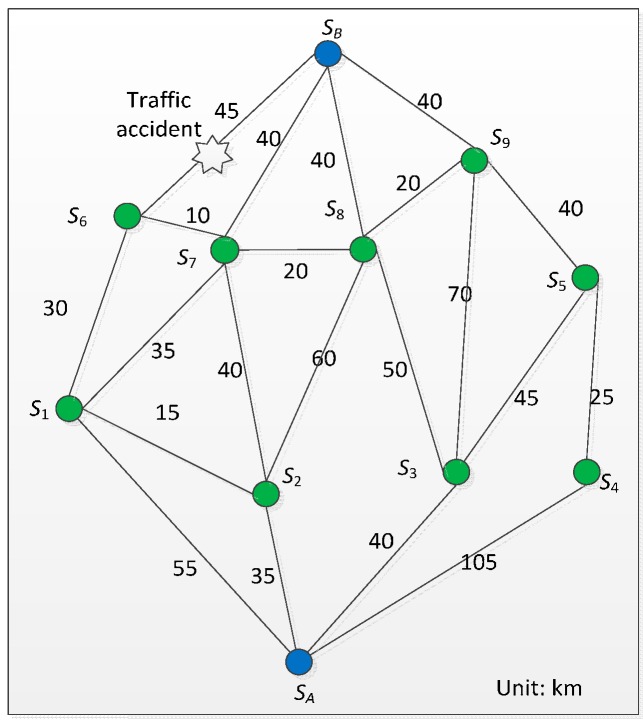
The distance of route segments from *A* to *B*.

**Figure 5:**
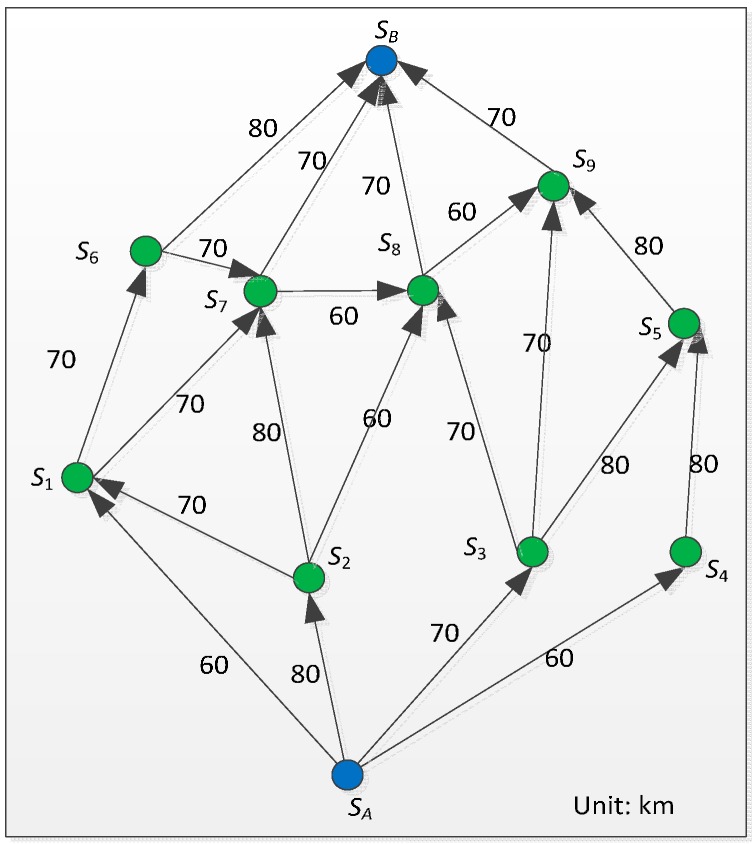
The average speed and direction of road segments from *A* to *B*.

In this paper, in order to give a quantitative analysis for the proposed approaches, we make some assumptions: (1) the distance from *A* to *B* is assumed to be known exactly (see Figure 4); (2) vehicles maintain a steady speed; and (3) the delay incurred by unexpected accidents is about 80 min. In Figure 4, nodes (e.g., *S*_1_) express landmarks for travel time aggregation. For the tensor regression approach, we could further assume that there is an RSE unit every ten kilometers deployed at strategic locations to exchange information with OBE installed on vehicles passing by. The direction and the average speed of each road segment are shown in Figure 5. We used MATLAB/Simulink as the simulation environment.

Table 1 shows the weight of routings with α=0.5, β=0.8 and γ=0. Table 2 shows the optimum path selection result for different locations of traffic accidents. For example, when a traffic accident occurs in the road segment between *S*_1_ and *S_B_*, vehicles cannot use this segment. We then set the distance between *S*_1_ and *S*_B_ to be infinite, and so the optimum path *S_A_ ➔ S*_1_ *➔ S_B_* between *S*_A_ and *S*_B_ is obtained.

**Table 1:** MCS weights for routing.

	α	β	Case 1		Case 2		Case 3		Case 4	
			Event	Weight	Event	Weight	Event	Weight	Event	Weight
S_A_-S_1_	0.5	0.8	0	44	0	44	0	44	0	44
S_A_-S_2_	0.5	0.8	0	28	0	28	0	28	∞	∞
S_A_-S_3_	0.5	0.8	0	32	0	32	0	32	∞	∞
S_A_-S_4_	0.5	0.8	0	84	0	84	0	84	0	84
S_1_-S_6_	0.5	0.8	0	24	0	24	0	24	0	24
S_1_-S_7_	0.5	0.8	0	28	0	28	0	28	0	28
S_2_-S_1_	0.5	0.8	0	12	0	12	∞	∞	0	12
S_2_-S_7_	0.5	0.8	+∞	∞	∞	∞	∞	∞	0	32
S_2_-S_8_	0.5	0.8	0	48	0	48	0	48	0	48
S_3_-S_8_	0.5	0.8	0	40	0	40	0	40	0	40
S_3_-S_9_	0.5	0.8	0	56	0	56	0	56	0	56
S_3_-S_5_	0.5	0.8	0	36	0	36	0	36	0	36
S_4_-S_5_	0.5	0.8	0	20	0	20	0	20	0	20
S_5_-S_9_	0.5	0.8	0	32	0	32	0	32	0	32
S_6_-S_B_	0.5	0.8	0	36	0	36	0	36	∞	∞
S_6_-S_7_	0.5	0.8	0	8	0	8	0	8	0	8
S_7_-S_B_	0.5	0.8	0	32	0	32	0	32	0	32
S_7_-S_B_	0.5	0.8	0	16	∞	∞	0	16	0	16
S_8_-S_B_	0.5	0.8	0	32	0	32	0	32	0	32
S_8_-S_9_	0.5	0.8	0	16	0	16	0	16	0	16
S_9_-S_B_	0.5	0.8	0	32	0	32	0	32	0	32

**Table 2:** Results of optimum path selection for different traffic accidents.

Case	Accident	Traditional	VANET	MCS
Case 1	*S*_2_ *➔ S_7_*	*S_A_ ➔ S_2_ ➔ A_S2-S7_ ➔ S_2_ ➔ S_1_ ➔ S_7_ ➔ S_B_*	*S_A_ ➔ S_2_ ➔ S_1_ ➔ S_7_ ➔ S_B_*	*S_A_ ➔ S_2_ ➔ S_1_ ➔ S_7_ ➔ S_B_*
Case 2	*S*_2_ *➔ S_7_ and S_7_ ➔ S_B_*	*S_A_ ➔ S_2_ ➔ A_S2-S7_ ➔ S_2_ ➔ S_1_ ➔ S_7_ ➔ A_S7-SB_ ➔ S_7_ ➔ S_8_ ➔ S_B_*	*S_A_ ➔ S_2_ ➔ S_1_ ➔ S_7_ ➔ S_8_ ➔ S_B_*	*S_A_ ➔ S_3_ ➔ S_8_ ➔ S_B_*
Case 3	*S*_2_ *➔ S_7_ and S_2_ ➔ S_1_*	*S_A_ ➔ S_2_ ➔ A_S2-S7_ ➔ S_2_ ➔ A_S2-S1_ ➔ S_2_ ➔ S_8_ ➔ S_B_*	*S_A_ ➔ S_2_ ➔ S_8_ ➔ S_B_*	*S_A_ ➔ S_3_ ➔ S_8_ ➔ S_B_*
Case 4	*S_A_ ➔ S_2_ and S_A_ ➔ S_3_ and S_6_ ➔ S_B_*	*S_A_ ➔ A_SA-S2_ ➔ S_A_ ➔ A_SA-S3_ ➔ S_A_ ➔ S_1_ ➔ S_6_ ➔ A_S6-SB_ ➔ S_6_ ➔ S_7_ ➔ S_B_*	*S_A_ ➔ S_1_ ➔ S_6_ ➔ S_7_ ➔ S_B_*	*S_A_ ➔ S_1_ ➔ S_7_ ➔ S_B_*

**Figure 6:**
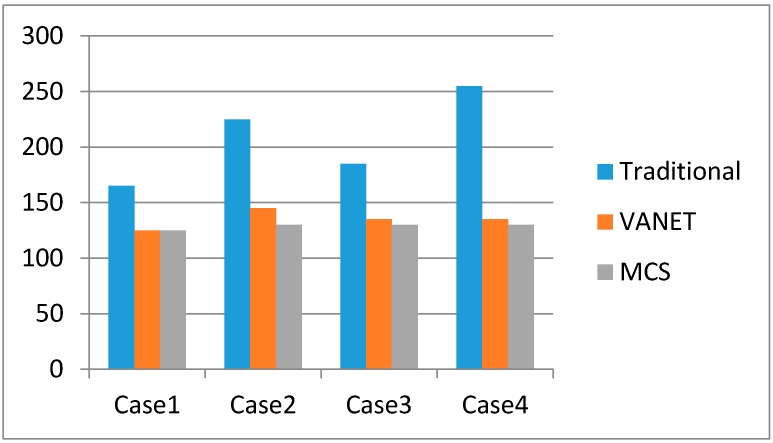
The distance travelled in the different cases.

**Figure 7:**
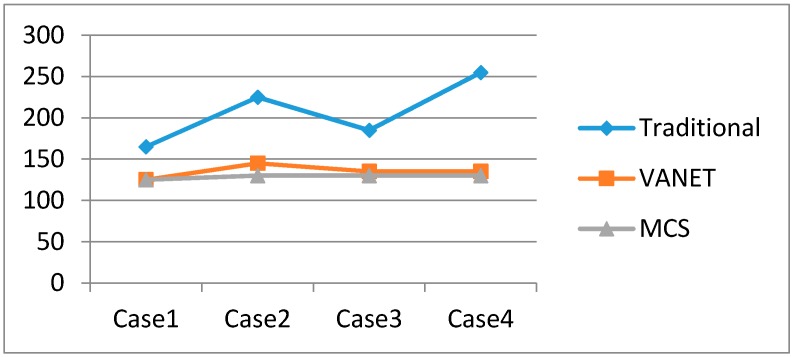
Time taken in the different cases.

As mentioned, we make use of MCS technology to support dynamic route choices for drivers. The distances and associated route times of different cases are also given in Figure 4. As we can see from Table 2, the timing of the traffic accident affects the routing choice. Figure 6 and Figure 7 show the validation of different vehicle routings in four example cases for avoiding traffic congestion. For case 2, without prediction, the vehicle will come across a sudden traffic accident between *S*_1_ and *S_B_*, which will result in an inevitable delay.

In this experiment, we made several assumptions to implement a quantitative analysis. Since the information about traffic accidents can be obtained from the traffic cloud, we can carry out dynamic route calculations periodically. In our view, the assumptions will not affect validation of the algorithm. Generally, the MCS approach has better real-time performance compared to the VANET method. 

From the simulation results, we can see that the planning algorithm based on MCS has some advantages over the others: (a) Quicker responsiveness. Taking Case 1 for example, the MCS-based planning algorithm can easily avoid the accident at *S*_2_ *➔ S_7_*, as it covers all the roads’ status in real-time, so its responsive speed is quicker than others; (b) Wider coverage. The CAIV can obtain more information from a larger number of smart phones simultaneously, so road status coverage can extend to many districts and even many cities. In Case 2, the algorithm based on MCS can select the proper route and avoid the congestion present in the initial phase of the route. Its coverage is wider than the traditional planning algorithm and the VANET-based planning algorithm.

### 5.2. Experiment

**Figure 8:**
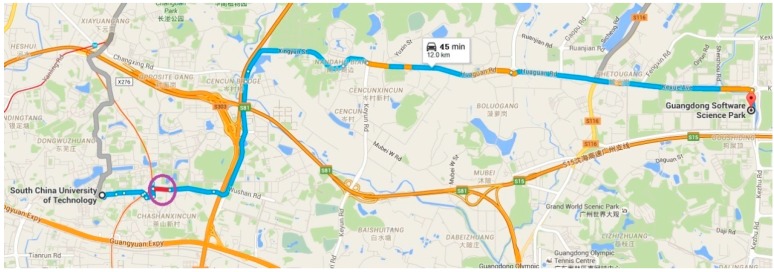
Route planning based on traditional algorithm.

**Figure 9:**
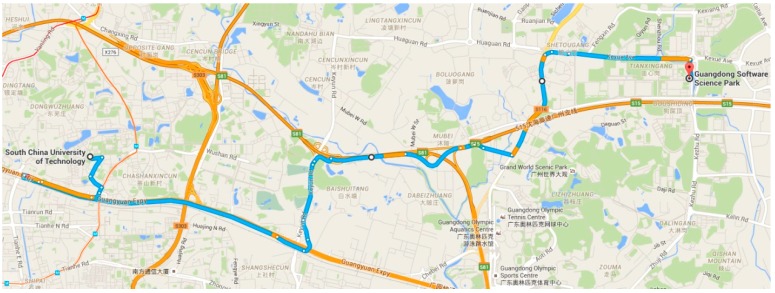
Route planning based on MCS.

We conducted an experiment to verify our algorithm in real road conditions. As shown in Figure 8, the starting point was the South China University of Technology, and the end point was Guangdong Software Science Park, marked as red. A traffic accident is indicated by the purple circle, where traffic flow is slow. Using the traditional route planning algorithm, the vehicle cannot avoid traffic congestion, and there are 11 traffic lights on the route selected. The distance travelled was 13 km, while the travel time was 45 min. Using the new route planning based on MCS, (Figure 9) the travel distance is 18 km, but the travel time is 25 min. On the way, we only encountered three traffic lights and avoid the traffic accident area. MCS technology can sense the traffic status more accurately and more quickly, and is therefore superior to the traditional method.

From the experimental results, it is easy to see the difference in effectiveness between route planning based on the traditional algorithm and that based on MCS. The algorithm based on MCS utilizes the real-time vehicular velocity and event information as criteria to select the optimal route and avoid congestion. The travel time is shortened from 45 min to 25 min, although the distance is 5 km longer than that of the route chosen by the traditional algorithm. This algorithm is appropriate for city traffic management, especially during rush hours, and can provide effective guidance to drivers.

## 6. Discussion and Outlook

In this paper, we study three approaches of dynamic route choice support for drivers to avoid congestion. In Table 3, we provide a qualitative comparison of all the studied approaches of congestion control. 

**Table 3:** A qualitative comparison among the three kinds of traffic congestion alleviation approaches.

Methods	Reliability	Service Contents	Interactions	Cloud
Travel time aggregation (using loop detectors and road-side cameras)	Low	Limited	V2I	VTC
Tensor regression approach (using VANETs)	Medium	Abundant	V2V, V2I	VTC, VAC
Mobile crowd sensing technology	High	Very abundant	V2V, V2I, V2H, and V2S	VWC

We also outline some insights for these traffic prediction approaches as follows:

*Travel Time Aggregation for Traffic Prediction*: We note that the vehicle’s information about the current conditions will typically be incomplete. It will virtually always deviate from the current traffic situation to some extent (e.g., because the situation changes over time). The route calculated by the VANET-based system may therefore be even worse than the standard route. The travel time benefit is thus highly dependent upon the dissemination performance: it will be large if up-to-date information relevant for the route calculation is available to the vehicle. It seems obvious that a larger number of RSUs improves the dissemination process performance and hence higher travel time savings can be achieved. This kind of static sensing is affected by several drawbacks, such as insufficient node coverage, high installation/maintenance cost, and lack of scalability.

*Tensor Regression Approach for Traffic Prediction*:Given real-time and accurate traffic information, each driver will typically select the best route in terms of minimum travel time, distance or other criteria. Intuitively, these decisions will collectively result in a state of Dynamic User Equilibrium (DUE). However, for a distributed system, where drivers make their own independent decisions based on the same travel time information, this may likely lead to a state similar to a Dynamic All-or-Nothing (DAN) assignment, since drivers with the same origin and destination will probably choose the same routes [32,33]. It is well known that the transportation network’s performance is optimal when the system is in a state of Dynamic System Optimal (DSO). Therefore, a decentralized and proactive dynamic vehicle routing algorithm should allow drivers to self-organize traffic and shift the system state from either DAN or DUE to DSO.

*MCS Technology for Traffic Prediction*: Traffic data collected by smartphones or sensor-equipped vehicles on the road, combined with the support of the traffic cloud where data mining takes place, make mobile sensing a versatile transportation system that can often replace traditional approaches (*i.e.*, static sensing infrastructures), and allow the realization of a broad range of applications. In order to attract prospective users’ attention and participation, we should adopt an incentive mechanism by considering the relationship between contributions and feedback services. The urban transportation system of Guangzhou was selected as an example for traffic prediction. We all know that the intelligence and mobility of the drivers can be leveraged to collect higher quality or semantically complex data. For example, drivers can easily identify traffic congestion levels and report them using pictures or text messages. According to the importance of area, the incentive mechanism should be designed by considering the weighting factors of each contribution [34]. However, the unavoidable reality is that we should pay close attention to privacy issues. For example, participants naturally have privacy concerns and personal preferences, and users may not want to share sensor data that contains or reveals private and sensitive information such as their current location.

## 7. Conclusions

In recent years, the emerging technologies (e.g., mobile cloud computing) together with the improvement of the infrastructure have brought new opportunities for traffic prediction and congestion alleviation. In this paper, we focus on two aspects: the taxonomy of CAIV and reliable traffic prediction approaches. The architecture of CAIV is divided into three primary architecture types: VTC, VAC, and VWC. Then, we briefly review traditional traffic prediction realized through both V2I and V2V communications. Subsequently, we propose a mobile crowd sensing technology to support dynamic route choices for drivers to avoid congestion. We also carry out experiments to verify the proposed approach. Finally, discuss the outlook of reliable traffic prediction. We believe that traffic prediction through cloud-assisted IoV will attract enormous attention and research efforts in the near future.
